# Catastrophic misinterpretation of bodily sensations and external events in panic disorder, other anxiety disorders, and healthy subjects: A systematic review and meta-analysis

**DOI:** 10.1371/journal.pone.0194493

**Published:** 2018-03-20

**Authors:** Barnabas Ohst, Brunna Tuschen-Caffier

**Affiliations:** Department of Clinical Psychology and Psychotherapy, Institute of Psychology, University of Freiburg, Freiburg, Germany; Brown University, UNITED STATES

## Abstract

The catastrophic misinterpretation model of panic disorder (PD) predicts that the catastrophic misinterpretation of bodily sensations is a distinctive characteristic of PD. Existing research on this prediction has produced mixed findings. This paper presents a systematic review and meta-analysis of studies comparing the strength of catastrophic misinterpretation of bodily sensations and external events in patients with PD, patients with other anxiety disorders, and healthy controls. Following a systematic screening, seven studies were included in the meta-analysis. For the catastrophic misinterpretation of bodily sensations, analyses showed medium to large effects between patients with PD and healthy controls and between patients with PD and patients with other anxiety disorders. For the catastrophic misinterpretation of external events, analyses showed medium to large effects between patients with PD and healthy controls and a small negative effect between patients with PD and patients with other anxiety disorders. The findings support the assumption that the catastrophic misinterpretation of bodily sensations is a distinctive characteristic of panic disorder and thus lend support to the catastrophic misinterpretation model of PD.

## Introduction

Catastrophic misinterpretation plays a central role in the cognitive model of panic disorder [1]. It is assumed that panic attacks result from the interpretation of (per se) harmless bodily sensations as signs of an impending physical, mental, or social catastrophe (e.g., interpreting a pounding heart as sign of an upcoming heart attack). Hence, the catastrophic misinterpretation of bodily sensations is assumed to be the cognitive process that is causally responsible for the emergence of fear that ultimately leads to a panic attack. It is further assumed that the catastrophic misinterpretation of bodily sensations is an enduring characteristic of patients with panic disorder (PD) and can therefore be measured in non-panic situations.

These assumptions distinguish the cognitive theory from another prominent theory of panic disorder, the alarm theory [2,3]. The alarm theory postulates that a panic attack is the occurrence of fear in a situation that does not actually pose a threat (“false alarm”). The repeated occurrence of such false alarms can lead to the conditioning of fear to internal cues (i.e., bodily sensations). Subsequently, the perception of bodily sensations can trigger the conditioned fear, thus leading to a panic attack. According to the alarm theory, a process of catastrophic misinterpretation is not assumed to take place. Instead, catastrophic cognitions (rather than catastrophic misinterpretations) are assumed to be an epiphenomenon of panic and to play no causal role in the emergence of panic [4].

### Catastrophic misinterpretation and catastrophic cognition

Both catastrophic misinterpretation and catastrophic cognition have been found to occur in a wide range of mental disorders (for a comprehensive review, see [5]). The present meta-analysis focuses on catastrophic misinterpretation in contrast to the closely related concept of catastrophic cognition. We follow the idea that catastrophic misinterpretation is a cognitive process and catastrophic cognition a possible result of that process [6,7]. We will briefly outline the distinguishing aspects of both concepts.

Catastrophic cognition describes acute thoughts [6] about an impending catastrophe, e.g., “I will have a heart attack”. Such thoughts are common in patients with PD or agoraphobia [8], but also occur in patients with other anxiety disorders (e.g., social anxiety disorder and generalized anxiety disorder; [9]).

Catastrophic misinterpretation describes the act of interpreting a stimulus as a sign of an impending catastrophe, for example, “My pounding heart means that I will have a heart attack” [1]. In this case, a given situation (i.e., feeling one’s heart pounding) is actively interpreted as signaling something else (i.e., having a heart attack). Therefore, catastrophic misinterpretation describes a cognitive process rather than a single thought. To the best of our knowledge following a systematic search and screening of publications concerned with catastrophic misinterpretation, the only instrument to measure this cognitive process is the Bodily Sensations Interpretation Questionnaire (BSIQ) in its various versions [10–12]. In the following, the term Bodily Sensations Interpretation Questionnaire and the acronym BSIQ are used as a generic term for all versions of this measure. If the version by Clark et al. [11] is meant, this is made explicit.

### Existing research on catastrophic misinterpretation

The cognitive model of panic predicts that the catastrophic misinterpretation of bodily sensations is more pronounced in patients with PD than in healthy controls and patients with other anxiety disorders. However, findings so far have been mixed. Only Clark et al. [11] have found the catastrophic misinterpretation of bodily sensations to be stronger in patients with PD than in healthy controls as well as in patients with other anxiety disorders (i.e., social anxiety disorder and generalized anxiety disorder) for all relevant outcome variables. For the comparison between patients with PD and healthy controls, several studies have found a difference in the strength of catastrophic misinterpretation of bodily sensations [10,12–15]. However, in none of these studies was this difference found on all outcome variables. For the comparison between patients with PD and patients with other anxiety disorders, Harvey et al. [13] found a difference in the strength of catastrophic misinterpretation of bodily sensations for one outcome variable, while Austin and Richards [12] and Austin and Kiropoulos [15] could not find a difference.

The cognitive model of PD makes no specific predictions concerning the catastrophic misinterpretation of external events. For the comparison between patients with PD and healthy controls, findings so far have been mixed. While some studies have found patients with PD to make more catastrophic misinterpretations of external events than healthy controls [10,11], others did not find this difference [14,15]. Comparing patients with PD and patients with other anxiety disorders, most studies have found no difference in the strength of catastrophic misinterpretation of external events [12,13].

The question of whether the catastrophic misinterpretation of bodily sensations is a distinctive characteristic of patients with PD is important due to its consequences for the focus of treatment. Following the cognitive model, treatment would focus on re-structuring the cognitions underlying the catastrophic misinterpretation [16]. Following the alarm theory, treatment would focus on extinction or counterconditioning exposure to relevant interoceptive cues [4]. Additionally, several strategies to enhance the effect of exposure therapy have been proposed [17]. Though existing research on catastrophic misinterpretation in patients with PD has produced mixed results, it is noteworthy that in some studies the sample size was small (e.g., [10]: n per group = 9; [13]: n per group = 12), decreasing the power of statistical analyses and increasing the probability of false negatives. The present paper therefore aims to review the evidence on whether the catastrophic misinterpretation of bodily sensations is a distinctive characteristic of patients with PD, focusing on effect sizes rather than on reported significance rates.

## Methods

### Search and selection strategy

A systematic search for articles on the strength of catastrophic misinterpretation in patients with PD compared to healthy controls and/or patients with other anxiety disorders was conducted by the first author in three major databases (PsycINFO, PubMed, and MEDLINE) on February 18, 2016 using the following search term: (“catastrophic interpretation” OR “catastrophic belief” OR “catastrophizing” OR “misinterpretation”) AND (“panic” OR “panic disorder” OR “panic attack” OR “anxiety attack”). The search was updated on February 8, 2017. As elaborated earlier, the present meta-analysis focuses on the cognitive process of catastrophic misinterpretation rather than catastrophic cognitions. Therefore, the term “catastrophic cognition” was not included in the search term. Papers were selected for inclusion if they were (1) empirical studies (2) investigating catastrophic misinterpretation (3) in patients with panic disorder (4) compared to healthy controls and/or patients with other anxiety disorders.

### Screening procedure

**Fig 1:**
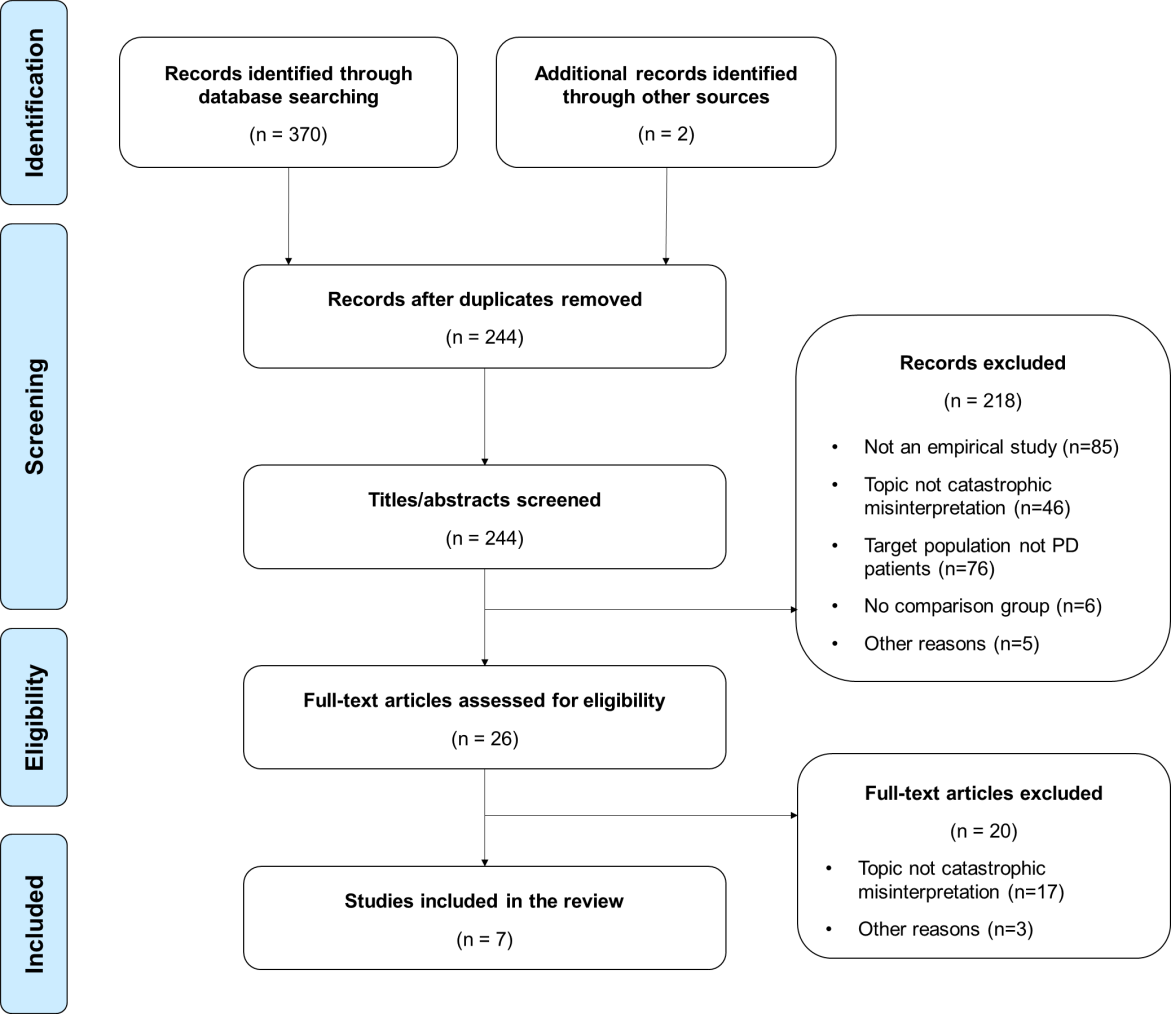
Flowchart outlining the search and selection strategy.

The initial search yielded 370 results (Fig 1). An additional two papers were identified through manual searches. After the removal of duplicates, 244 records remained. Based on the screening of titles and abstracts, 218 papers were excluded because (1) they were not an empirical study (e.g., case studies, reviews), (2) the topic was not catastrophic misinterpretation (e.g., catastrophic cognitions as symptom), (3) the sample did not include patients with PD, (4) there was no comparison group (i.e., healthy controls and/or patients with other anxiety disorders), or (5) other reasons (e.g., not available in English). Following an initial review of the remaining 26 papers, 20 papers were excluded because (1) the topic was not catastrophic misinterpretation or (2) other reasons (e.g., not original data). Therefore, seven studies were included in the present meta-analysis (Table 1). Note that since Clark et al. [11] reported two relevant studies in one paper, seven studies but only six papers were included.

**Table 1:** Basic characteristics of studies included in the meta-analysis.

Study	Country	Sample	CM measure
McNally & Foa 1987 [10]	USA	N = 27 (9 PD, 9 NCP, 9 HC)	IQ
Harvey, Richards, Dziadosz, & Swindell 1993 [13]	Australia	N = 36 (12 PD, 12 SAD, 12 HC)	IQ
Clark et al. 1997, Study 1 [11]	UK	N = 60 (20 PD, 20 SAD/GAD, 20 HC)	BSIQ
Clark et al. 1997, Study 2 [11]	Sweden	N = 196 (45 PD, 106 SAD/GAD, 45 HC)	BBSIQ
Richards, Austin, & Alvarenga 2001 [14]	Australia	N = 114 (20 PD, 25 NCP, 69 HC)	BBSIQ
Austin & Richards 2006 [12]	Australia	N = 113 (38 PD, 21 SAD, 20 NCP, 34 HC)	BSIQ-M
Austin & Kiropoulos 2008 [15]	Australia	N = 88 (30 PD, 28 SAD, 30 HC)	BSIQ-M

Citations are listed in order of year of publication. CM = Catastrophic Misinterpretation; PD = Panic Disorder; SAD = Social Anxiety Disorder; GAD = Generalized Anxiety Disorder; NCP = Non-Clinical Panickers; HC = Healthy Controls; IQ = Interpretation Questionnaire [10]; BSIQ = Bodily Sensations Interpretation Questionnaire [11]; BBSIQ = Brief Bodily Sensations Interpretation Questionnaire [11]; BSIQ-M = Bodily Sensations Interpretation Questionnaire-Modified [12].

### The bodily sensations interpretation questionnaire (BSIQ)

In all seven included studies, a version of the BSIQ was used as the measure of catastrophic misinterpretation. Table 1 shows which version was used in each study.

Each version of the BSIQ can be described in the following general terms. The items measure so-called internal events (i.e., bodily sensations) and external events. External events include general events (e.g., smelling smoke) and social events (e.g., being ignored by a shop assistant). Each item consists of two parts. In the first part, participants are presented with a situation (e.g., “You notice that your heart is beating quickly and pounding.”) and are asked to provide an explanation (“Why?”). The open-ended responses are coded as either harm-related (e.g., “I will have a heart attack.”), anxiety-related (e.g., “I will have a panic attack.”) or benign (e.g., “I did sports.”). Since the meaning of anxiety-related responses is disputed [11,18], the present meta-analysis focuses on harm-related responses as the outcome.

In the second part of each item, participants are presented with three potential explanations for the given situation (e.g., “Because you have been physically active.”) and are asked to rank them in the order in which they would be most likely to come into their mind in the given situation. In the IQ and the BSIQ by Clark et al. [11], one of these explanations is harm-related and two are benign, while in the BSIQ-M one benign option is replaced by an anxiety-related option. This modification of the task can lead to an artificially increased score for harm-related responses for healthy controls and patients with other anxiety disorders, thus diminishing the difference to patients with PD. Therefore, the scores for ranked responses of studies using the BSIQ-M are not included in the overall effects reported in this meta-analysis.

### Data extraction

Scores for open-ended and ranked harm-related responses were extracted. The score for harm- and anxiety-related open-ended responses was omitted, since its meaning is still under dispute. Since only two studies using the BSIQ-M reported the newly introduced outcome response, this score was omitted as well. All scores are reported separately for bodily sensations and external events. The data was extracted by the first author.

### Statistical analyses

All statistical analyses were conducted with Stata version 14.1 [19]. To determine effect sizes, Hedges' *g* was calculated from reported means and standard deviations. This was done for the effects between patients with PD and healthy controls and between patients with PD and patients with other anxiety disorders. Hedges' *g* was chosen over Cohen’s *d* to correct for small sample sizes. Effect sizes are classified as small (*d* ≥ 0.2), medium (*d* ≥ 0.5), or large (*d* ≥ 0.8), in accordance with Cohen (1980). The heterogeneity of effect sizes across studies was estimated with the *I*^2^ index. A high *I*^2^ value (i.e., I2 > 75%; [20]) reflects different effect sizes across studies, a low *I*^2^ value (i.e., I2 < 50%; [20]) reflects similar effect sizes across studies.

### Risk of bias

As recommended in the PRISMA statement [21], the risk of bias in individual studies as well as across studies should be assessed for all studies included in a systematic review or meta-analysis. Concerning the risk of bias in individual studies, the assessment of risk should also be considered in data synthesis. However, recommended markers for the risk of bias in individual studies are fitted for randomized controlled trials (RCTs; [21]). The studies included in the present meta-analysis are exclusively questionnaire-based. Thus, only few of the recommended markers would apply to these studies and a consideration of these for data synthesis would give a lot of weight to these particular markers. Therefore, the authors decided not to assess the risk of bias in individual studies formally. Instead, potentially relevant aspects are discussed in the respective sections.

Concerning the risk of bias across studies, funnel plots [22] were created for each outcome measure (S9–S16 Figs), plotting the standardized mean difference (SMD) against the SMD’s standard error. An asymmetrical distribution of studies can be interpreted as indicator of a publication bias [23]. Formal tests of asymmetry, however, rely on a sufficient number of studies. For example, for the widely used Egger test for asymmetry at least 10 studies are recommended [24]. Therefore, due to the small number of papers included in this meta-analysis (*N* = 6), no statistical test of asymmetry was conducted.

## Results

### Catastrophic misinterpretation of bodily sensations

Concerning the catastrophic misinterpretation of bodily sensations, medium effects were found for open-ended responses when comparing patients with PD with healthy controls and with patients with other anxiety disorders (Table 2). For ranked responses, large effects were found comparing patients with PD with healthy controls and with patients with other anxiety disorders. It is noteworthy that the effects for ranked responses (2.09 and 1.24) were considerably larger than for open-ended responses (.76 and .60).

**Table 2:** Overall effect sizes concerning the catastrophic misinterpretation of bodily sensations and external events in comparison with patients with PD.

	**Healthy Controls**		**Other Anxiety Disorders**	
**Studies included**	6^a^	5^b^	4^a^	3^b^
**Response format**	Open-ended responses	Ranked responses	Open-ended responses	Ranked responses
	**Bodily sensations**			
**Overall effect size: Hedges' *g***	.76	2.09	.60	1.24
**95% Confidence Interval**	.51 to 1.01	1.77 to 2.42	.29 to .90	.97 to 1.51
**Heterogenity: *I*^2^**	61%	70%	28%	2%
	**External events**			
**Overall effect size: Hedges' *g***	.76	.93	-.06	-.41
**95% Confidence Interval**	.51 to 1.00	.66 to 1.20	-.36 to .24	-.66 to -.16
**Heterogenity: *I*^2^**	6%	36%	63%	88%

Clark et al. [11] reported separate scores for general and social external events in Study 1 and for SAD and GAD in Study 2. Since all other studies reported combined scores (i.e., “external events” and “other anxiety disorders”), for Clark et al. [11] effect sizes of both scales (Study 1) and of both anxiety disorder groups (Study 2) were combined.

^a^Study 2 by Clark et al. [11] is not included since only scores for ranked responses were reported.

^b^The two studies using the BSIQ-M are not included due to methodological reasons (see above). Their combined effect sizes are -.06 (CI: -.40 to .34) and .29 (CI: -.12 to .63) for bodily sensations and .37 (CI: .02 to .71) and -.61 (CI: -.99 to -.23) for external events comparing patients with PD with healthy controls and patients with other anxiety disorders, respectively.

### Catastrophic misinterpretation of external events

Concerning the catastrophic misinterpretation of external events, a medium effect was found for open-ended responses and a large effect for ranked responses comparing patients with PD and healthy controls. Between patients with PD and patients with other anxiety disorders, no effect was found for open-ended responses and a small negative effect for ranked responses.

## Discussion

The present paper aimed to explore the question of whether the catastrophic misinterpretation of bodily sensations is a distinctive characteristic of patients with PD. The overall effects reported in this meta-analysis corroborate this assumption. The finding that the catastrophic misinterpretation of bodily sensations was found to be more pronounced in patients with PD than in patients with other anxiety disorders lends particularly strong support to this assumption. Therefore, the catastrophic misinterpretation of bodily sensations seems not to be a facet of catastrophizing that is common to all patients with anxiety disorders [25]. Rather, it seems to be a distinctive characteristic of patients with PD. This finding argues against the assumption that the catastrophic cognitions found in patients with PD (e.g., “I will have a heart attack.”) are merely an epiphenomenon of panic, as postulated by proponents of the alarm theory [4]. Rather, this finding supports the assumption that these catastrophic cognitions are the result of catastrophic misinterpretation.

As mentioned earlier, it is striking that for the catastrophic misinterpretation of bodily sensations, the effects for ranked responses (2.09 between patients with PD and healthy controls and 1.24 between patients with PD and patients with other anxiety disorders) were considerably larger than for open-ended responses (.76 and .60). An explanation for this discrepancy was discussed by Harvey et al. [13]. They proposed that the open-ended response format might not sufficiently activate relevant threat-related cognitive schemata responsible for the catastrophic misinterpretation of bodily sensations in patients with PD. Therefore, catastrophic misinterpretations that would be made in panic-relevant situations in everyday life are not made in the laboratory. If the BSIQ was administered under circumstances that might facilitate the process of catastrophic misinterpretation (e.g., by experimentally activating relevant threat-related cognitive schemata), the catastrophic misinterpretation of bodily sensations might show more clearly in open-ended responses among patients with PD. Further research is required to investigate this issue.

Concerning the catastrophic misinterpretation of external events, the cognitive model of PD makes no specific predictions. The pattern of effects found in this meta-analysis supports the assumption that patients with an anxiety disorder can be expected to be more inclined to interpret ambiguous situations in a catastrophic fashion than healthy subjects [25]. Both the finding that patients with PD clearly showed more catastrophic misinterpretations of external events than healthy subjects (medium to large effects), and the finding that there was only a small difference between patients with PD and patients with other anxiety disorders (no effect for open-ended responses and a small negative effect for ranked responses) are in line with this explanation.

However, concerning the pattern of effects found for external events between patients with PD and patients with other anxiety disorders, it is noteworthy that the level of anxiety for both groups was not assessed in all included studies, even though it can be expected to have an influence on this outcome. Three studies assessed state anxiety ([13]; [11], Study 1 and 2), one study additionally assessed trait anxiety [13], one study assessed anxiety within the past week [15], and one study did not assess anxiety [12]. When anxiety was assessed, only Harvey et al. found a siginifcant difference between both groups (i.e., concerning trait anxiety) with patients with SAD scoring higher than patients with PD [13]. However, they also found the largest effect (.45) of all included studies for ranked responses for external events, even though one could have expected a small or none effect due to the higher trait anxiety for patients with SAD. For open-ended responses for external events, Harvey et al. found a small effect (.19), which is in-between the effects found by the other two studies by Clark et al. ([11], Study 1: .66 and Study 2: -.71), in both of which no significant difference between both groups cocerning state anxiety was observed. In the authors’ opinion, no valid conclusion concerning the influence of differences in the level of anxiety between patients with PD and patients with other anxiety disorders on the catastrophic misinterpretation of external events can be drawn based on the existing data. However, this does naturally not mean, that there is no systematic influence. To be able to investigate such a potential systematic influence, it would be helpful if future studies assessed all relevant types of anxiety (i.e., state anxiety, trait anxiety, and anxiety sensitivity) using identical and well-validated instruments.

There are several limitations to this meta-analysis that need to be considered. First, the number of studies included was rather small. This leads to a high impact of any single study and can hence lead to an over- or under-estimation of effects by chance findings in single studies. More research on catastrophic misinterpretation in patients with PD and patients with other anxiety disorders is called for. Second, the included studies used different versions of the BSIQ. The variation in items may have had an impact on participants’ scores and thus on the effects reported in this meta-analysis. Because of the small number of studies available, it was not possible to obtain more homogeneity, for example by only including studies using the same version of the BSIQ.

## Conclusions

To the authors’ knowledge, this is the first meta-analysis for research on catastrophic misinterpretation in patients with PD. While the results suggest that the catastrophic misinterpretation of bodily sensations is a distinctive characteristic of patients with PD and thus lend support to the cognitive model of PD, more research, especially on the role of catastrophic misinterpretations in the emergence of panic, is required to extend the understanding of panic and to develop improved PD-specific treatments.

## Supporting information

S1 File PRISMA checklist. (DOCX)

S1 Fig Forest plot for SMD between patients with PD and healthy controls for open-ended responses concerning bodily sensations. (TIF)

S2 Fig Forest plot for SMD between patients with PD and patients with other anxiety disorders for open-ended responses concerning bodily sensations. (TIF)

S3 Fig Forest plot for SMD between patients with PD and healthy controls for ranked responses concerning bodily sensations. (TIF)

S4 Fig Forest plot for SMD between patients with PD and patients with other anxiety disorders for ranked responses concerning bodily sensations. (TIF)

S5 Fig Forest plot for SMD between patients with PD and healthy controls for open-ended responses concerning external events. (TIF)

S6 Fig Forest plot for SMD between patients with PD and patients with other anxiety disorders for open-ended responses concerning external events. (TIF)

S7 Fig Forest plot for SMD between patients with PD and healthy controls for ranked responses concerning external events. (TIF)

S8 Fig Forest plot for SMD between patients with PD and patients with other anxiety disorders for ranked responses concerning external events. (TIF)

S9 Fig Funnel plot for SMD and standard error of SMD between patients with PD and healthy controls for open-ended responses concerning bodily sensations. (TIF)

S10 Fig Funnel plot for SMD and standard error of SMD between patients with PD and patients with other anxiety disorders for open-ended responses concerning bodily sensations. (TIF)

S11 Fig Funnel plot for SMD and standard error of SMD between patients with PD and healthy controls for ranked responses concerning bodily sensations. (TIF)

S12 Fig Funnel plot for SMD and standard error of SMD between patients with PD and patients with other anxiety disorders for ranked responses concerning bodily sensations. (TIF)

S13 Fig Funnel plot for SMD and standard error of SMD between patients with PD and healthy controls for open-ended responses concerning external events. (TIF)

S14 Fig Funnel plot for SMD and standard error of SMD between patients with PD and patients with other anxiety disorders for open-ended responses concerning external events. (TIF)

S15 Fig Funnel plot for SMD and standard error of SMD between patients with PD and healthy controls for ranked responses concerning external events. (TIF)

S16 Fig Funnel plot for SMD and standard error of SMD between patients with PD and patients with other anxiety disorders for ranked responses concerning external events. (TIF)
