# Supplementary figures and images for: Catastrophic misinterpretation of bodily sensations and external events in panic disorder, other anxiety disorders, and healthy subjects: A systematic review and meta-analysis
Source: PLoS One. 2018 Mar 20;13(3):e0194493. doi: 10.1371/journal.pone.0194493 (PMC5860765; doi:10.1371/journal.pone.0194493)

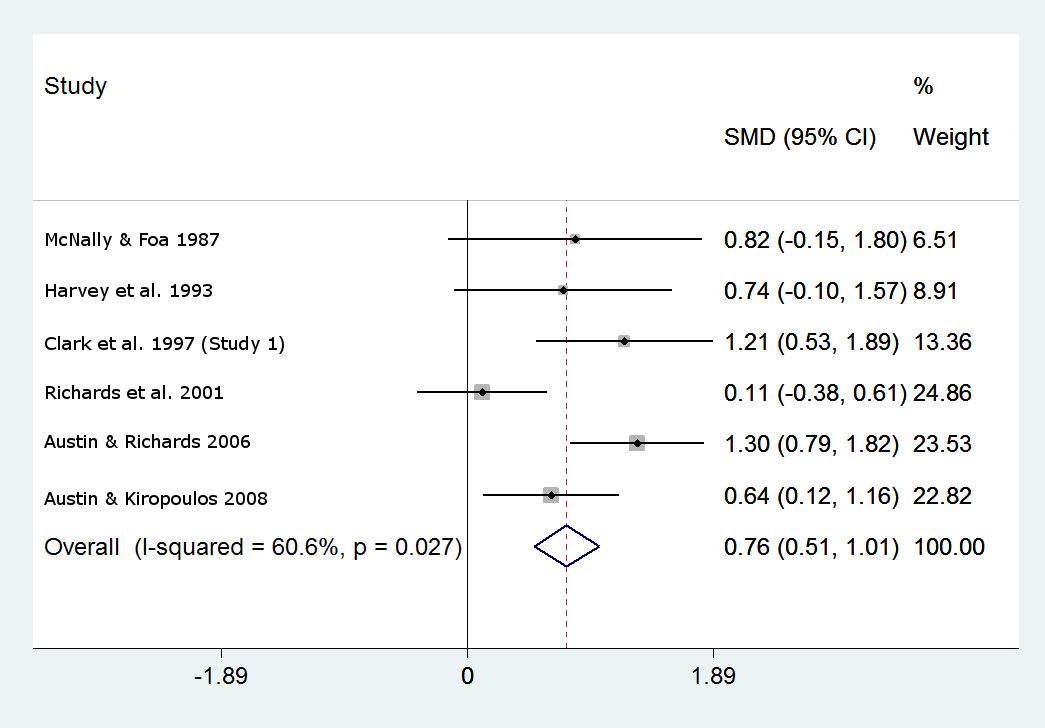

Supplement: S1 Fig — (TIF) [file pone.0194493.s002.tif]

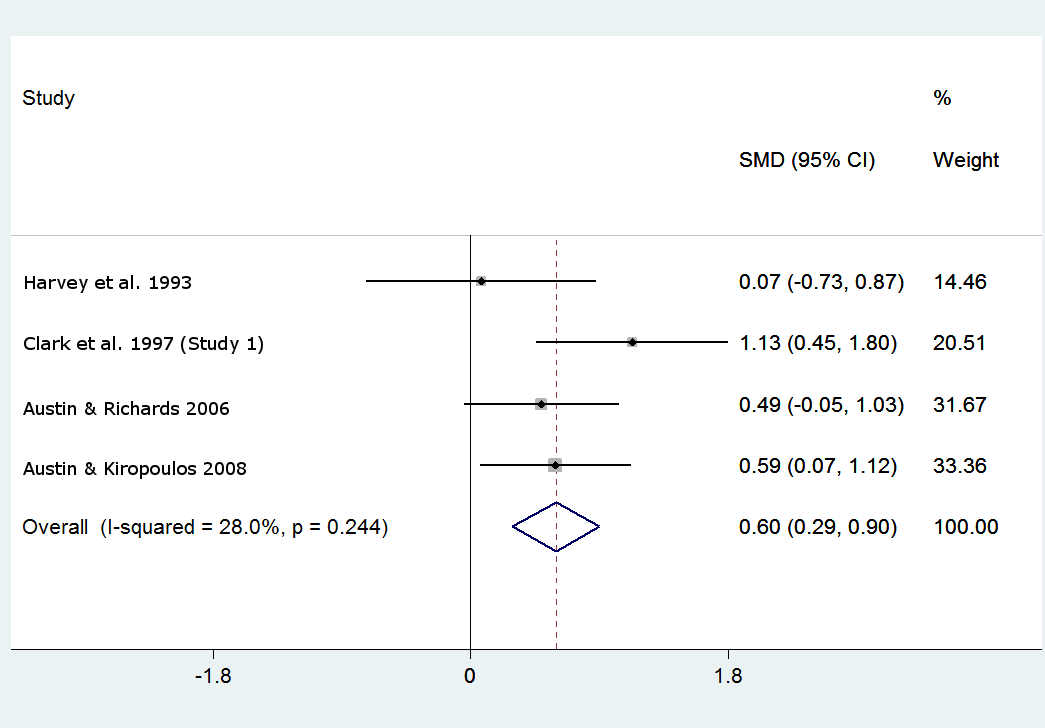

Supplement: S2 Fig — (TIF) [file pone.0194493.s003.tif]

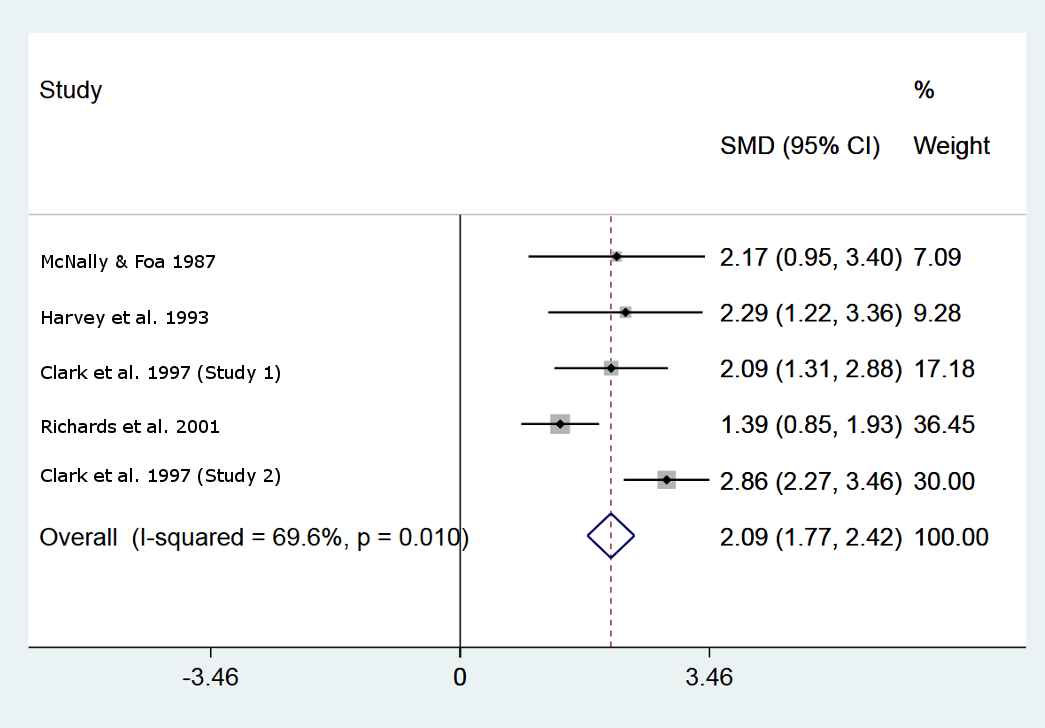

Supplement: S3 Fig — (TIF) [file pone.0194493.s004.tif]

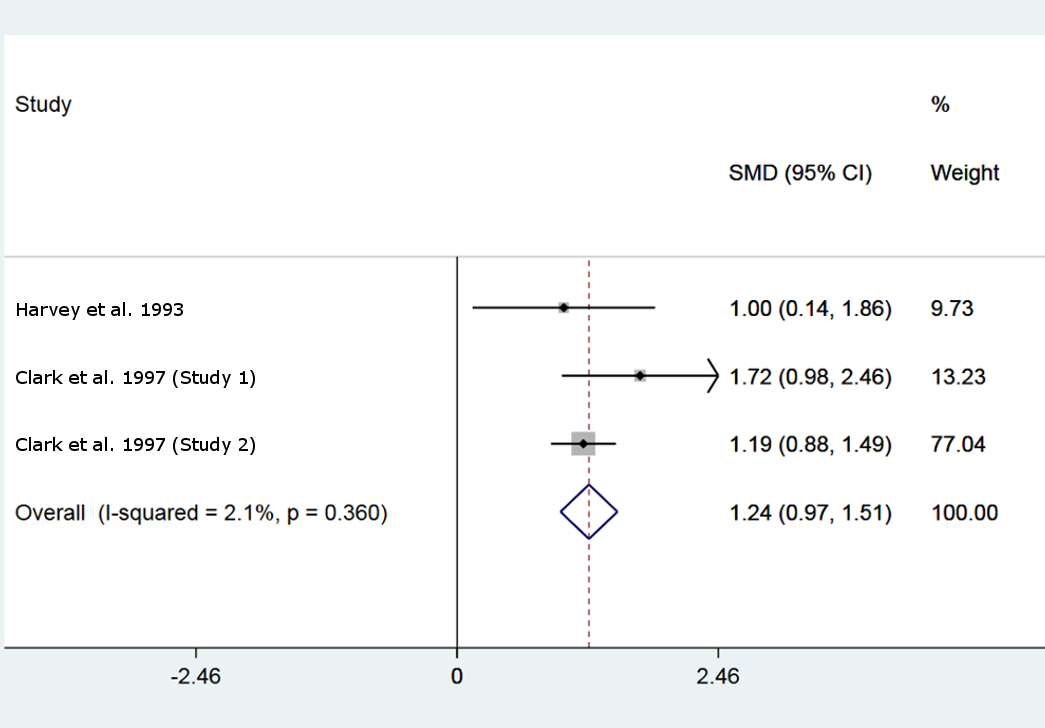

Supplement: S4 Fig — (TIF) [file pone.0194493.s005.tif]

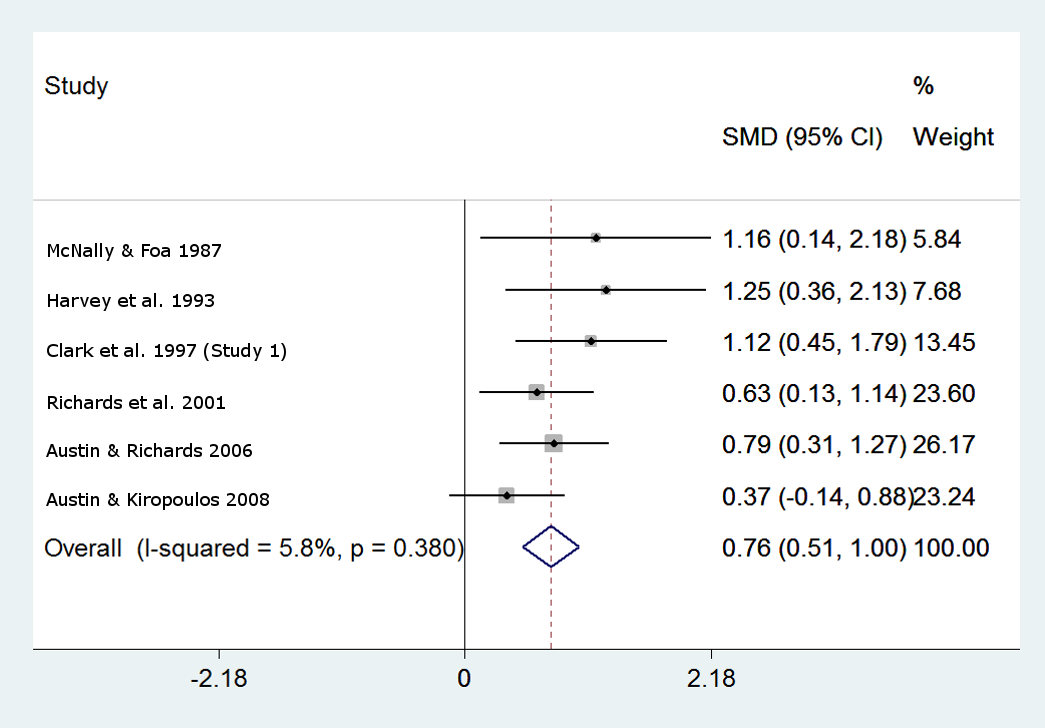

Supplement: S5 Fig — (TIF) [file pone.0194493.s006.tif]

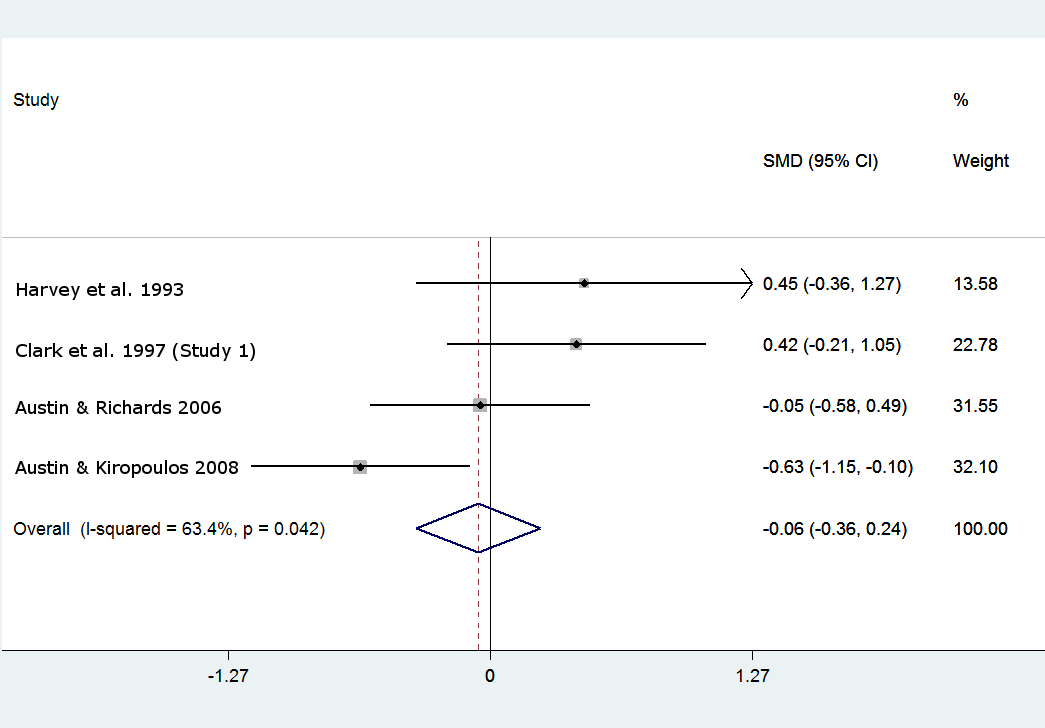

Supplement: S6 Fig — (TIF) [file pone.0194493.s007.tif]

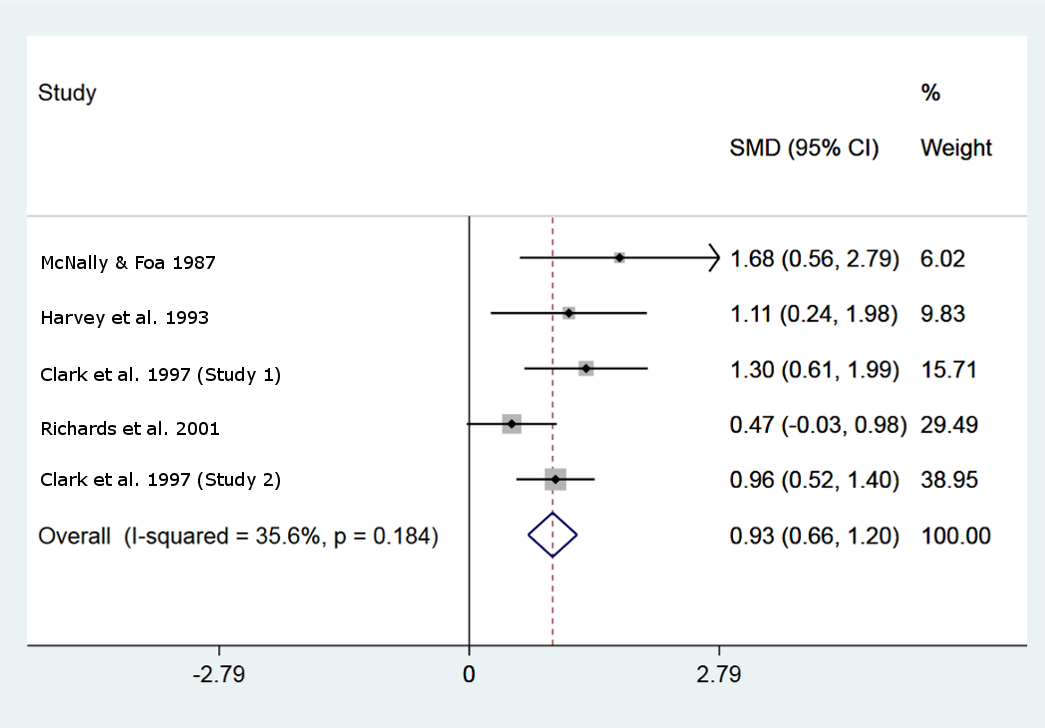

Supplement: S7 Fig — (TIF) [file pone.0194493.s008.tif]

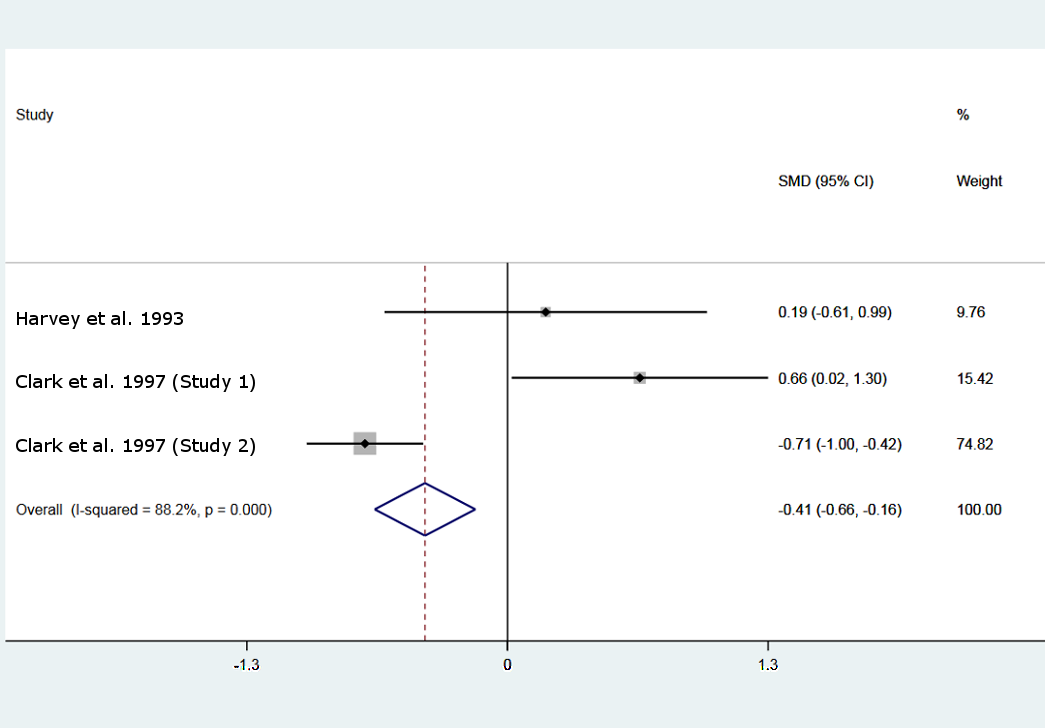

Supplement: S8 Fig — (TIF) [file pone.0194493.s009.tif]

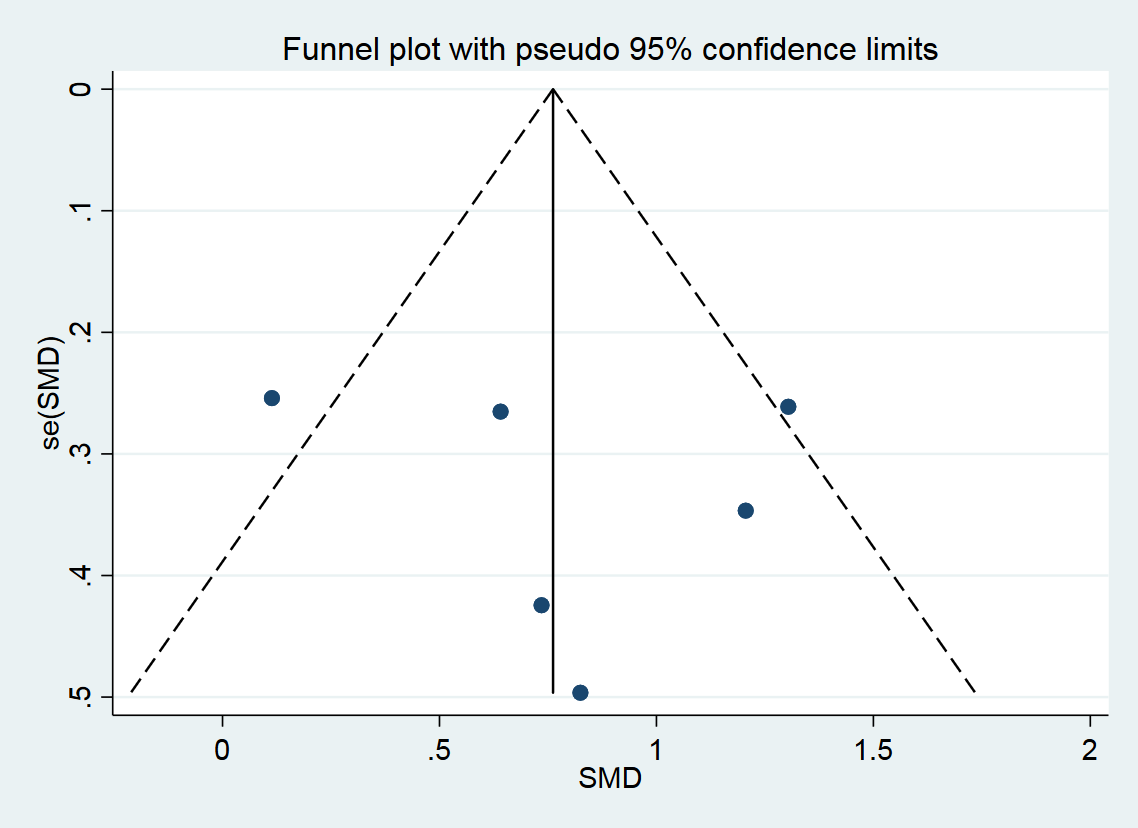

Supplement: S9 Fig — (TIF) [file pone.0194493.s010.tif]

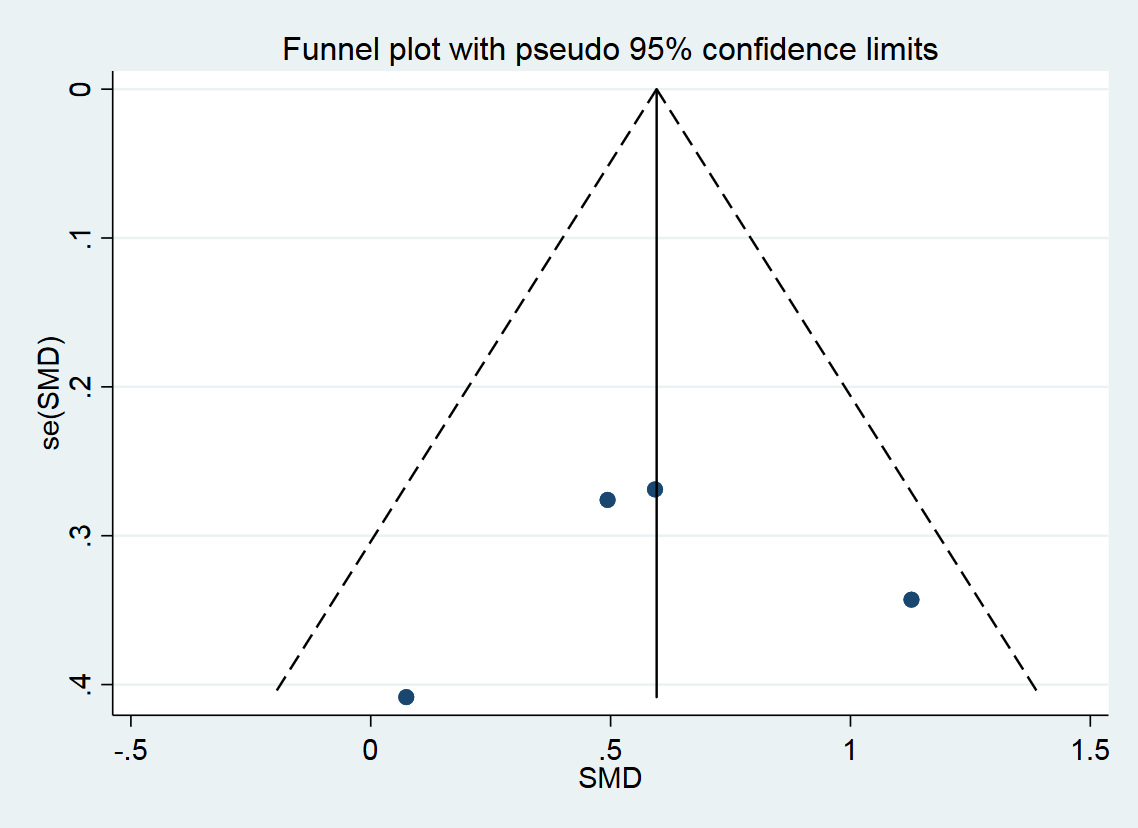

Supplement: S10 Fig — (TIF) [file pone.0194493.s011.tif]

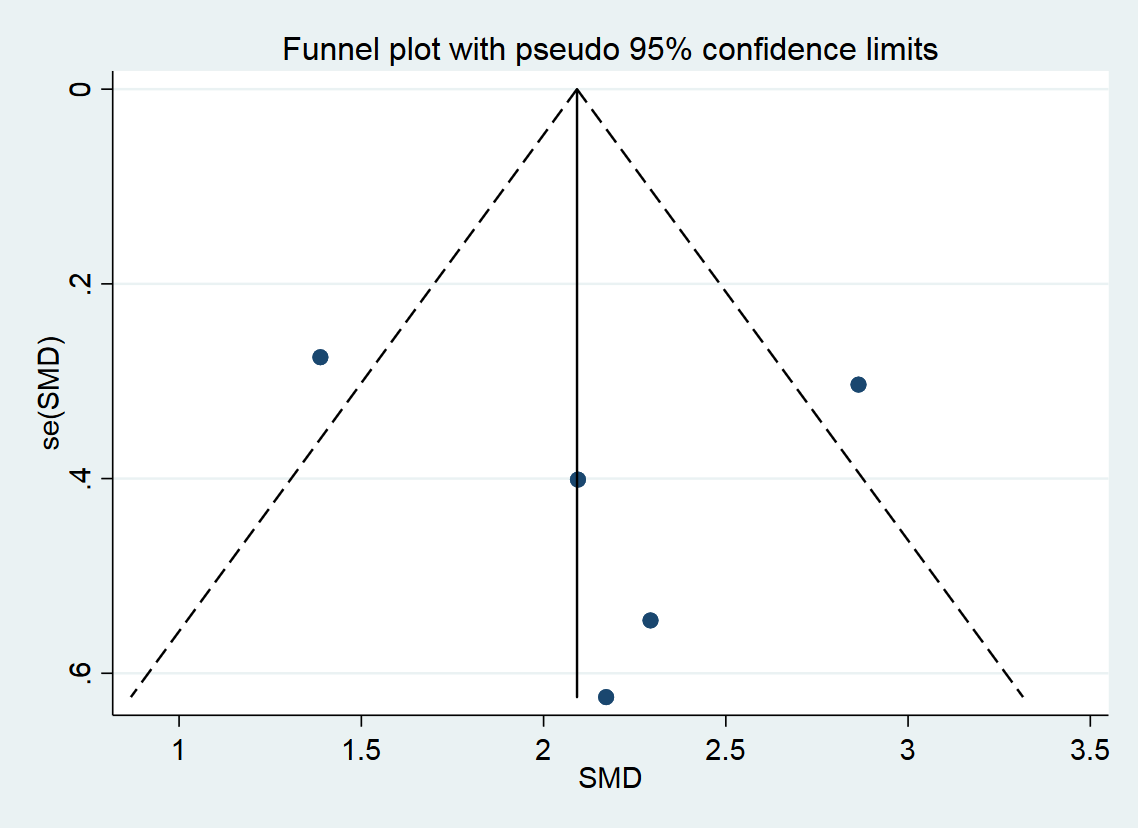

Supplement: S11 Fig — (TIF) [file pone.0194493.s012.tif]

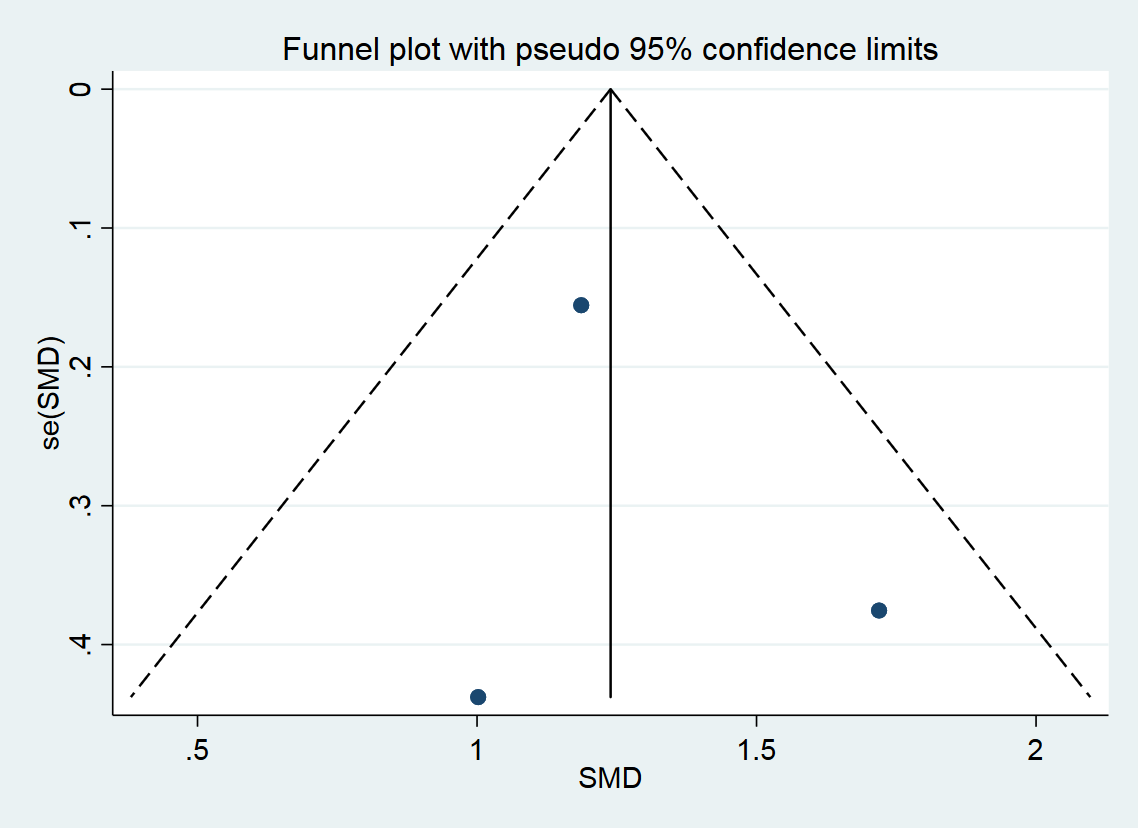

Supplement: S12 Fig — (TIF) [file pone.0194493.s013.tif]

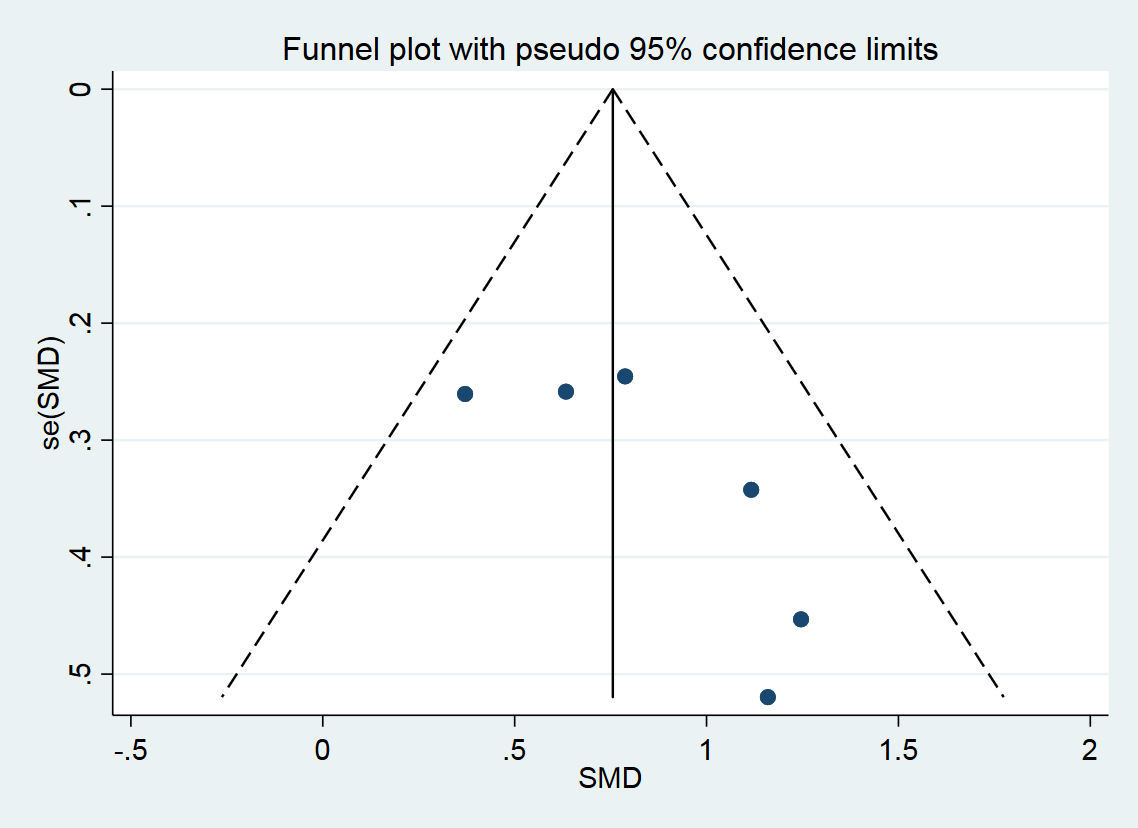

Supplement: S13 Fig — (TIF) [file pone.0194493.s014.tif]

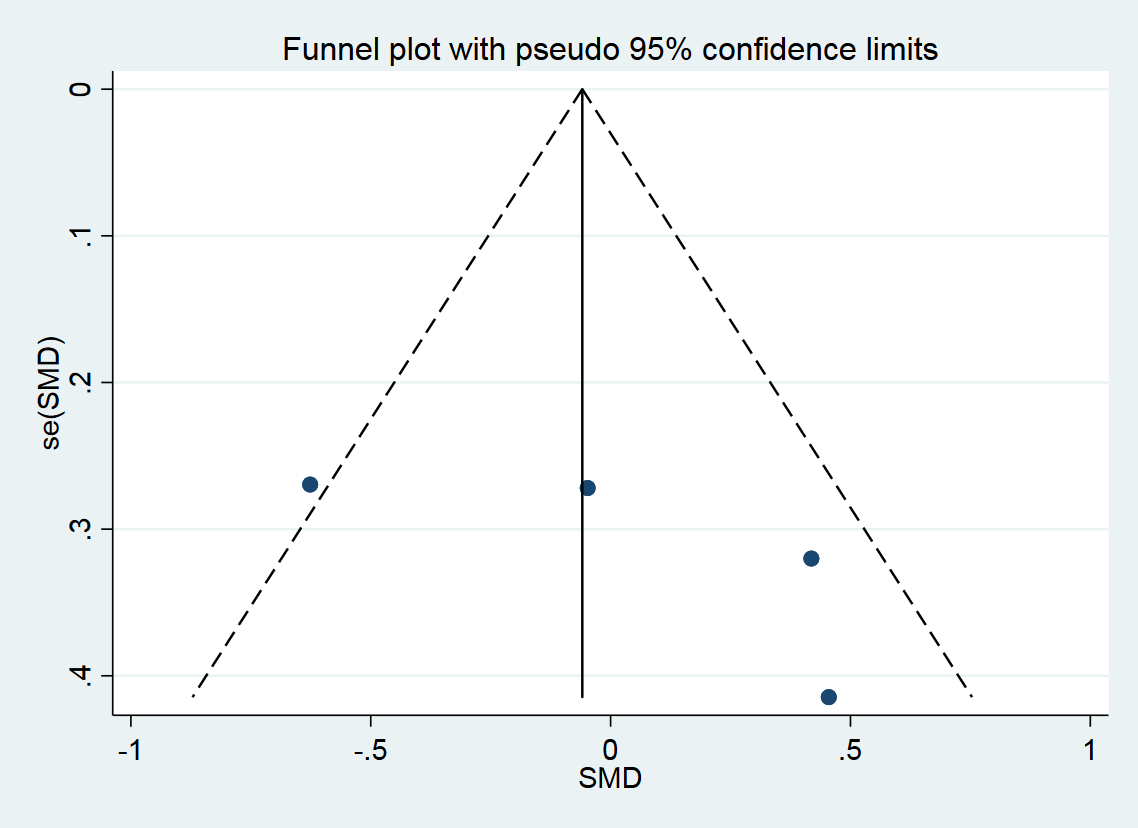

Supplement: S14 Fig — (TIF) [file pone.0194493.s015.tif]

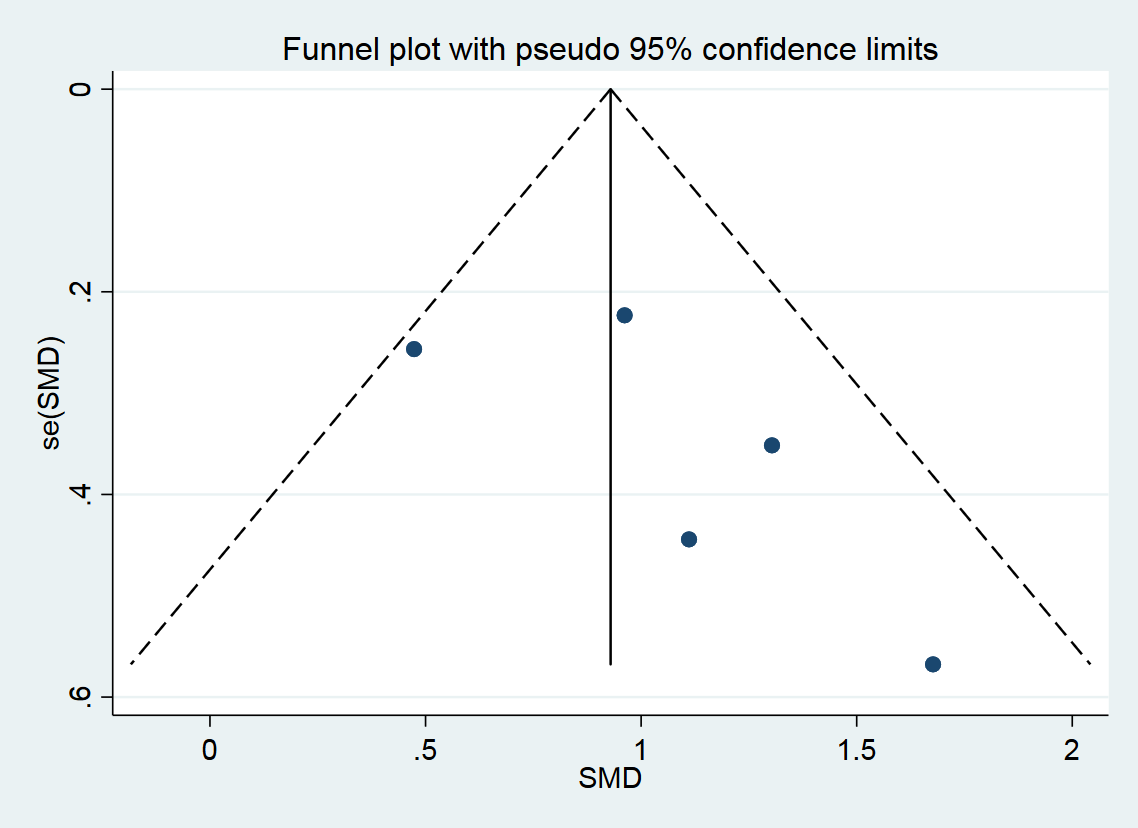

Supplement: S15 Fig — (TIF) [file pone.0194493.s016.tif]

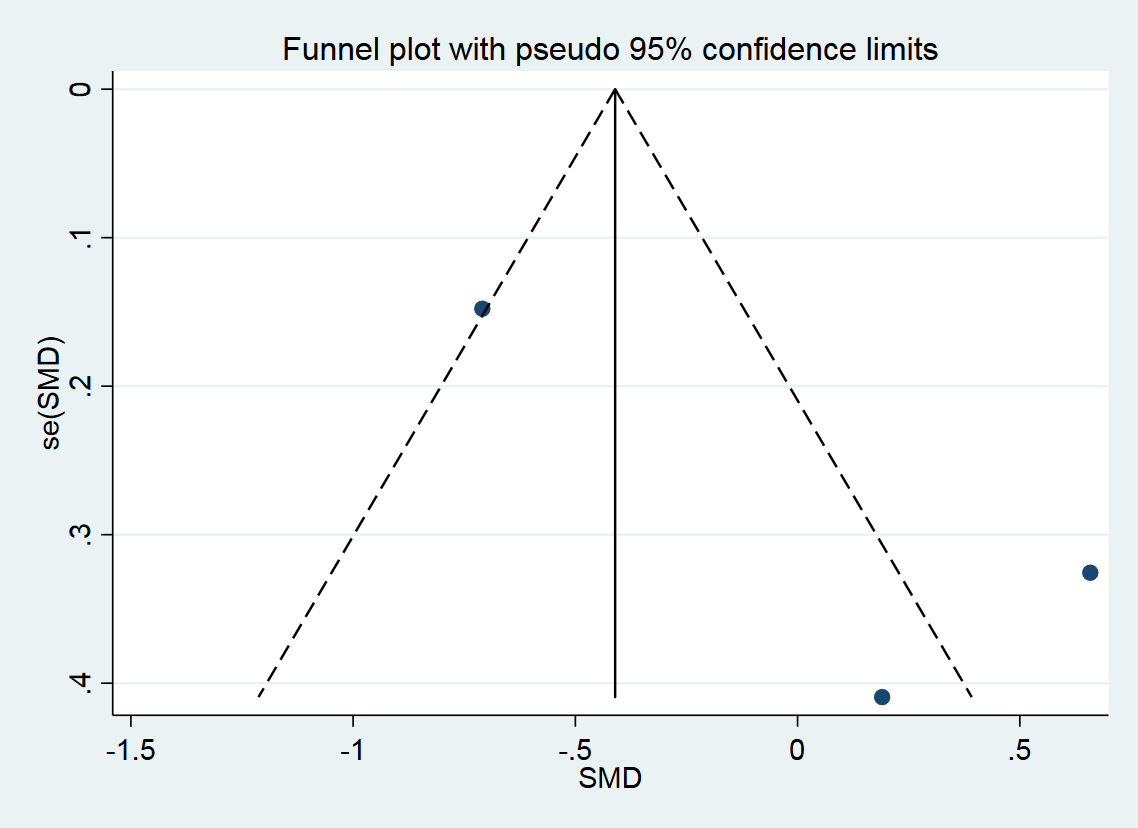

Supplement: S16 Fig — (TIF) [file pone.0194493.s017.tif]
